# Locating the Human Cardiac Conduction System Using a 3D Model of Its Nutritious Arteries

**DOI:** 10.1038/s41598-017-00504-1

**Published:** 2017-03-23

**Authors:** Yu Xu, Yukun Peng, Rongmei Qu, Guorong Zheng, Feiyan Feng, Yan Feng, Linying He, Shanli He, Zeyu Li, Chang Liu, Zhaoming Xiao, Jun Ouyang, Jingxing Dai

**Affiliations:** 1 First Clinical School, Southern Medical University, Guangzhou, China; 2 Department of Anatomy, Guangdong Provincial Medical Biomechanical Key Laboratory, Southern Medical University, Guangzhou, China; 3 Department of Radiology, Guangdong No. 2 Provincial People’s Hospital, Guangzhou, China; 4 Department of Electrocardiogram, Xinjiang Uygur Autonomous Region People’s Hospital, Urumqi, China; 5 Second Clinical School, Southern Medical University, Guangzhou, China

## Abstract

It is difficult for anatomists to dissect the human cardiac conduction system (CCS) on specimens as well as for cardiovascular clinicians to locate the CCS during cardiac operations. Here, we demonstrate a new method for locating the CCS using a 3D model of its nutritious arteries. First, we perfused the coronary arteries with contrast material and then acquired a set of data of thin computer tomography (CT) scans. Then, we generated a 3D model of the coronary artery and distinguished the arteries that supply the CCS. We then located the CCS on the 3D model via its nutritious arteries and dissected the CCS. Finally, the structures that were dissected were removed for histological and immunofluorescent staining. The results of histological and immunofluorescence examination proved the structure to be the CCS. Thus, we successfully located the CCS using a 3D model of its nutritious arteries. We suggest that with this new method, cardiac surgeons can locate a patient’s CCS during cardiac surgeries such as transcatheter aortic valve implantation (TAVI) or radiofrequency catheter ablation (RFCA).

## Introduction

The cardiac conduction system (CCS) is made up of several subcomponents, each of which plays a critical role in the heartbeat. The subcomponents of the CCS include the sinoatrial node (SAN), internodal bundle, atrioventricular node (AVN), His bundle branch (HBB), left bundle branch (LBB), right bundle branch (RBB) and Purkinje fibers (PFs). Due to the muscular origin of both the CCS and myocardium, visually distinguishing these two tissues is difficult, which causes great difficulty in identifying the CCS not only on gross specimens but also in surgeries.

At present, there are approximately three methods for the anatomical dissection of the CCS. The first is dissection of the CCS directly by the naked eye or under a dissecting microscope^1^. However, verification of whether the dissected structure is the CCS requires histological confirmation. Therefore, the accuracy of this method is not high. The second method was reported by Tawara, who found a fibrous sheath around part of the CCS that forms a system of interconnecting channels that can be injected with Indian ink^2^. However, Davies^3^ reported that this method cannot be used successfully in humans because in humans the sheath is excessively thin and cannot tolerate the increasing perfusing pressure. Damián Sánchez-Quintana^4^ and Andrew Atkinson^5^ reported that the sheath of human Purkinje fibers disappear at the ventricular apex. In addition, the sheath does not exist in the SAN, AVN and the proximal stump of the HBB^6^. Therefore, the complete structure of the CCS cannot be shown using this method. The third method uses glycogen, which is abundant in the human LBB and PFs. Uhley^7^ visualized the PFs with Lugol’s solution. However, the biggest limitation of this method is that the hearts must be examined shortly postmortem because glycogen disappears very rapidly after death. In addition to the time limitation, the glycogen content can be influenced by the type of the cardiac disease. There is no method that can help cardiac surgeons visually locate the CCS. For this reason, a simple and accurate method to locate the CCS is needed for anatomists and cardiovascular specialists.

The aim of this study is to find a new method to locate the CCS that can be applied to cardiovascular operations as well. Here, for the first time, we describe a new method: locating the CCS using a 3D model of its nutritious arteries.

## Results

**Figure 1:**
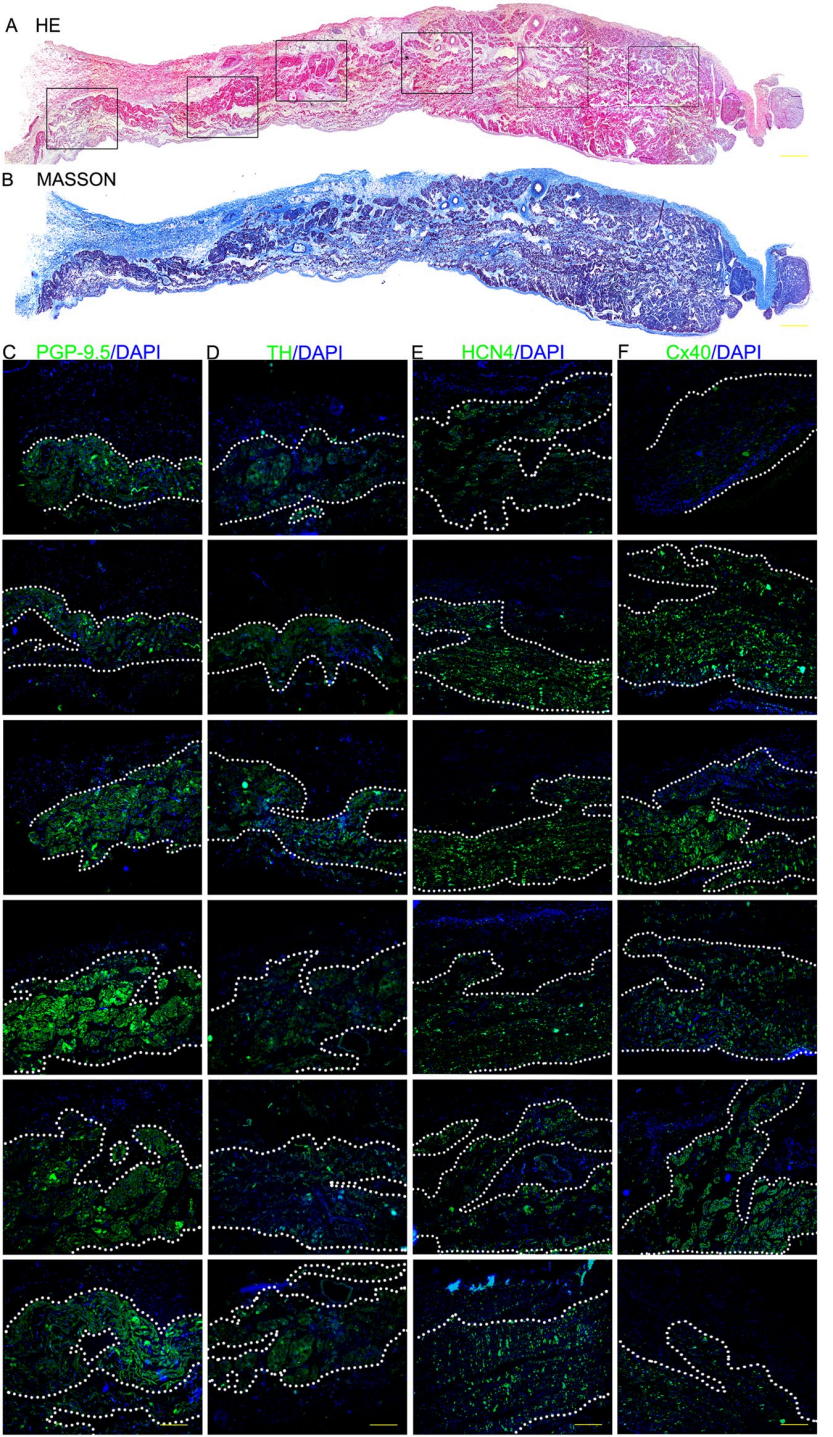
The results of the histological and immunofluorescence examination of the SAN. (**A)** HE staining (scale bar = 1200 μm). (**B**) Masson staining (scale bar = 1200 μm). (**C**) Immunofluorescence staining with the anti-protein gene product 9.5 antibody (PGP9.5, the green region is the positive area). (**D**) Immunofluorescence staining with the anti-tyrosine hydroxylase antibody (TH, the green region is the positive area). (**E**) Immunofluorescence staining with the anti-connexin 40 antibody (Cx40, the green region is the positive area). (**F**) Immunofluorescence staining with the anti-hyperpolarization-activated cyclic nucleotide-gated channel 4 antibody (HCN4, the green region is the positive area). In **C–F**, cell nuclei are stained with DAPI, scale bar = 400 μm.

**Figure 2:**
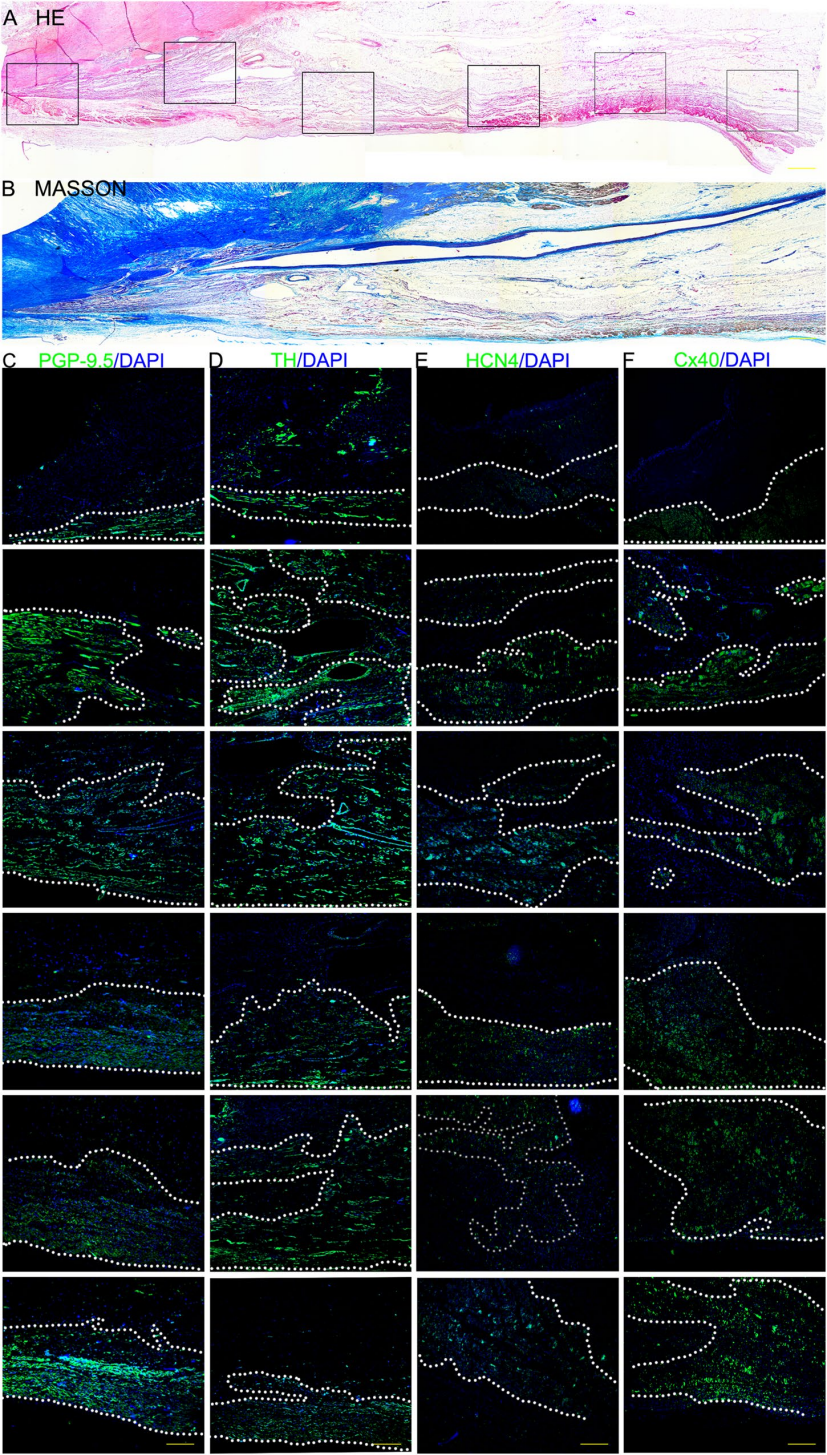
The results of the histological and immunofluorescence examination of the AVN. (**A**) HE staining (scale bar = 1200 μm). (**B**) Masson staining (scale bar = 1200 μm). (**C**) Immunofluorescence staining with the anti-PGP9.5 antibody. (**D**) Immunofluorescence staining with the anti-TH antibody. (**E**) Immunofluorescence staining with the anti-Cx40 antibody. (**F**) Immunofluorescence staining with the anti-HCN4 antibody. In **C–F**, the green region is the positive area, and the cell nuclei are stained with DAPI, scale bar = 400 μm.

**Figure 3:**
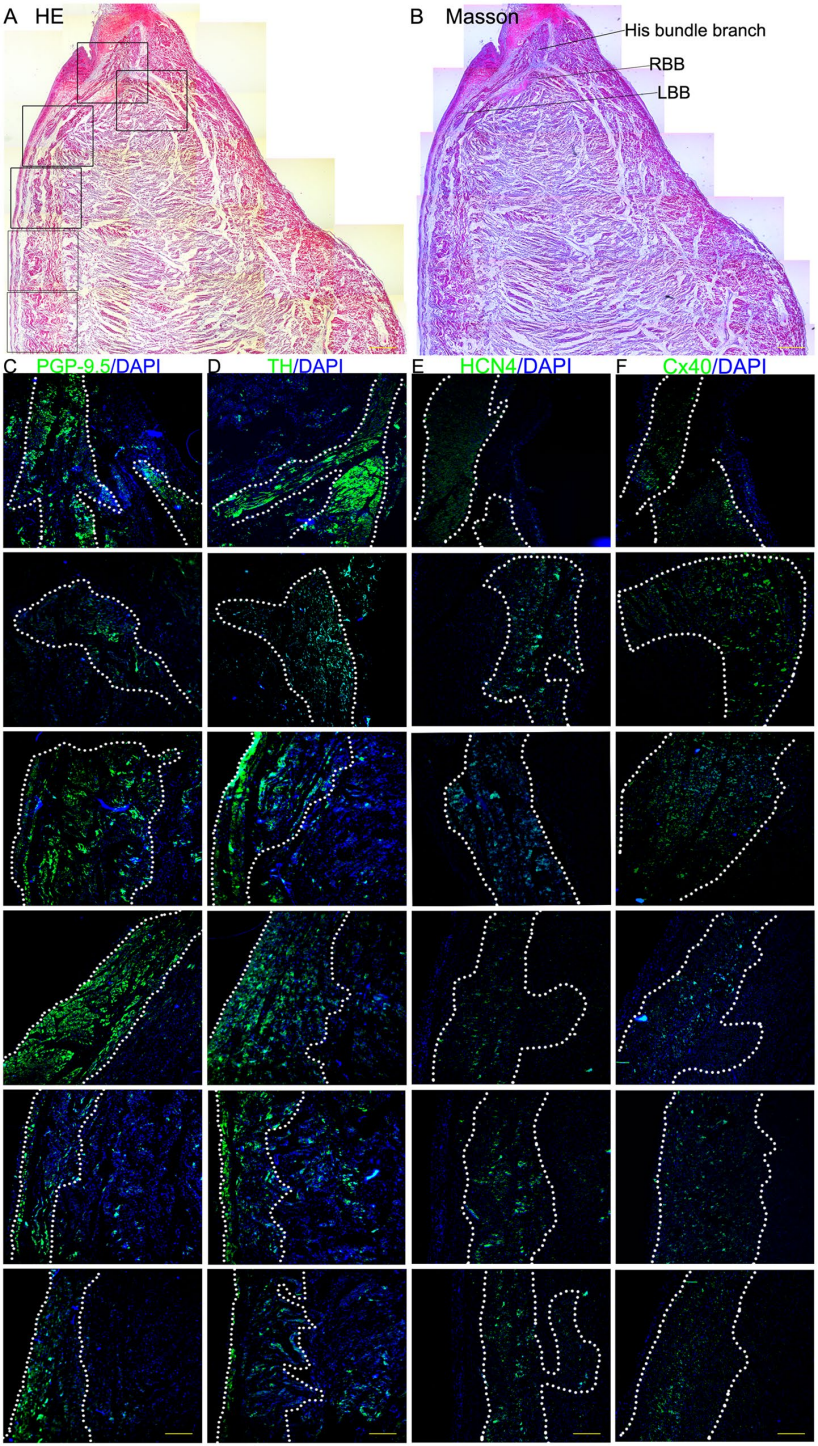
The results of the histological and immunofluorescence examination of the HBB and RBB. (**A**) HE staining (scale bar = 1200 μm). (**B**) Masson staining (scale bar = 1200 μm). (**C**) Immunofluorescence staining with the anti-PGP9.5 antibody. (**D**) Immunofluorescence staining with the anti-TH antibody. (**E**) Immunofluorescence staining with the anti-Cx40 antibody. (**F**) Immunofluorescence staining with the anti-HCN4 antibody. In **C–F**, the green region is the positive area, and the cell nuclei are stained with DAPI, scale bar = 400 μm.

**Figure 4:**
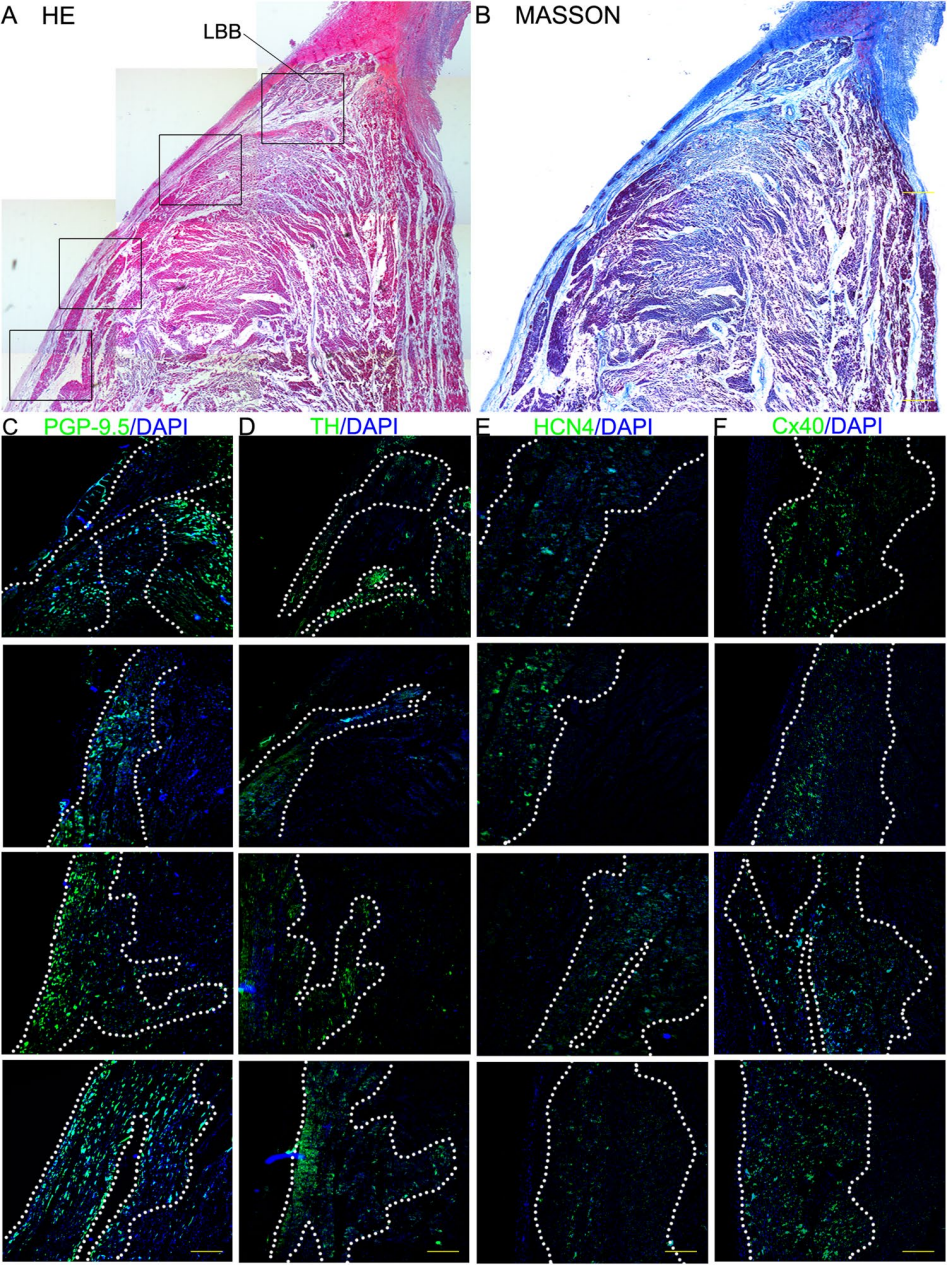
The results of the histological and immunofluorescence examination of the LBB. (**A**) HE staining (scale bar = 1200 μm). (**B**) Masson staining (scale bar = 1200 μm). (**C**) Immunofluorescence staining with the anti-PGP9.5 antibody. (**D**) Immunofluorescence staining with the anti-TH antibody. (**E**) Immunofluorescence staining with the anti-Cx40 antibody. (**F**) Immunofluorescence staining with the anti-HCN4 antibody. In **C–F**, the green region is the positive area, and the cell nuclei are stained with DAPI, scale bar = 400 μm.

The structures that we dissected using the 3D model of the nutritious arteries of the CCS were removed to undergo histological and immunofluorescent staining. The results of the histological and immunofluorescence examination are illustrated in Figs 1–4. In addition, the results of the Acetylcholine esterase (AChE) staining are illustrated in the supplemental files (Fig. S1).

In histological sections (Fig. 1), the SAN has a distinct border that can be observed between the node and the adjacent myocardium in HE and Masson staining. In immunofluorescence-stained sections, the SAN is the area positive for PGP9.5, TH, HCN4 and Cx40.

In histological sections (Fig. 2), we can observe the whole transition of the AVN; the node arranged in parallel and the branch of the node seem to project into the fiber. The AVN finally crosses into the HBB to traverse to the central fibrous body. In immunofluorescence-stained sections, the AVN and HBB are the areas positive for PGP9.5, TH, HCN4 and Cx40.

In histological sections (Fig. 3), the HBB is divided into the LBB and RBB. The HBB, LBB and RBB are insulated from the neighboring myocardium by fibrous sheaths. In immunofluorescence-stained sections, the HBB, LBB and RBB are the areas positive for PGP9.5, TH, HCN4 and Cx40.

In Fig. 4, the LBB is insulated from the neighboring myocardium by fibrous sheaths (in HE and Masson staining), and the LBB is the area positive for PGP9.5, TH, HCN4 and Cx40 in the immunofluorescence-stained sections.

## Discussion

In this research, we located the CCS on a 3D model using its nutritious arteries as a guide. We dissected the CCS per the location on the 3D model and then dissected the tissue for histological and immunofluorescence examination to verify the accuracy of this new method. Because the specimens have undergone long procedures (such as perfusion, CT scanning and anatomy dissection), the AChE staining was not very good. Therefore, we put these data in the supplemental files (Fig. S1). Per the histological and immunofluorescence results, we have learned that the accuracy of this method is higher than that of traditional methods.

This new method has significant importance in clinical application for cardiac surgeons. We can use the following procedures for some cardiac surgeries. First, perform an enhanced 320-row CT scan of the patient’s heart. Second, reconstruct the 3D model of the coronary artery. Third, identify the nutritious arteries of the CCS and then locate the CCS on the 3D model. In this way, we can know the location of the CCS and its nutritious arteries during cardiac operations to reduce the injury to the CCS. A high heart rate increases the motion artifact of coronary arteries and limits the image quality^8^. Multicycle reconstruction (MCR) is used for patients with high heart rates to improve the temporal resolution^9^. The temporal resolution of a 320-row CT scan is improved from 175 to 87 ms using MCR from two heart beats^10^. Lee *et al*.^11^ showed that with MCR in patients with heart rates over 65 beats per minute (bpm), a coronary artery image of diagnostic quality similar to that of patients with heart rates 65 bpm can be obtained.

Moreover, this new method can play a useful role in TAVI. TAVI is an effective means designed to treat elderly patients with severe aortic stenosis that are unsuitable for surgical aortic valve replacement. TAVI has been performed on more than 50,000 patients worldwide since 2002. However, there is still high-grade atrioventricular (AV) block associated with TAVI. Permanent pacemaker implantation is the most frequently reported complication of TAVI, and it occurred much more often than surgical aortic valve replacement (17.7% vs 4.0%)^12^. The rate of aortic valve regurgitation is also high in TAVI (14.0%)^13^. Tomokazu Kawashima^14^ noted that the morphology and location of the HBB and LBB are directly associated with the AV block after TAVI.

**Figure 5:**
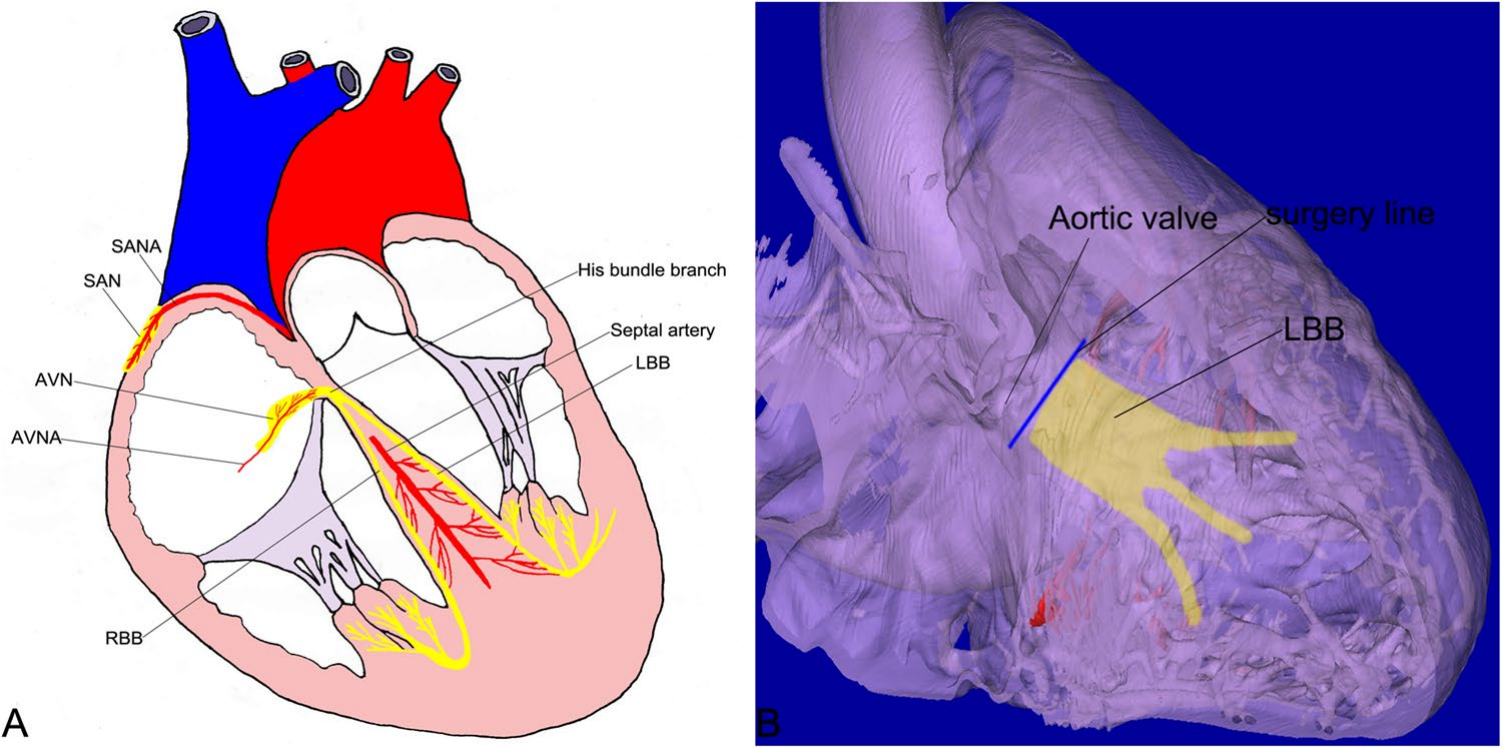
Diagram and illustration. (**A**) Diagram of the cardiac conduction system (CCS) and its nutritious arteries. (**B**) The 3D model illustrates that, during some surgeries, surgeons should pay attention to the location of the LBB and its nutritious arteries. In TAVI, surgeons cannot put the aortic prosthesis over the surgery line (in blue).

Regarding this new method, we can use the following procedures in TAVI. Before performing TAVI, an enhanced CT scan of the heart can be performed on the heart. On the one hand, surgeons can reconstruct the 3D model of the coronary artery, identify the nutritious arteries of the AVN, HBB and LBB, and then locate the AVN, HB and LBB on the 3D model. On the other hand, CT can provide crucial information about the aortic root. Cardiac surgeons can observe the cardiac valve calcification and assess the aortic prosthesis size for TAVI, which can reduce the rate of aortic valve regurgitation in TAVI. So, during the procedure, surgeons can be careful to avoid the CCS, place the aortic prosthesis in an appropriate location, and avoid placing the aortic prosthesis over the surgery line and crushing the LBB and its nutritious arteries (Fig. 5). In this way, the rate of AV block and aortic valve regurgitation after TAVI can be reduced.

## Materials and Methods

### Perfusion

For this study, we used 3 adult human hearts without any heart disease from donated cadavers at Southern Medical University. The study was approved by the Ethics Committee of the College of Basic Medicine, Southern Medical University and was performed per the Declaration of Helsinki. All body donors gave written consent to obtain and use their specimens for medical research or teaching. The right and left coronary arteries were cannulated with polyethylene tubes. After cannulation of the coronary arteries, 15 ml 10% formal solution was first injected by hand into each heart to prevent cell autolysis, which is good for histological and immunofluorescence examination. Then, the contrast material (a suspension of 60% latex rubber mixed with red pigment and 0.4 g/ml bismuth oxide) was injected into the right and left coronary arteries of each heart at a controlled pressure (90 to 110 mmHg, depending on the size of the heart) until the arteries and their branches were clearly visible to the naked eye at room temperature. The hearts were stuffed with cotton in every heart chamber to maintain their shape.

### Data acquisition and reconstruction of the 3D model

These hearts were scanned immediately with CT using a 0.8-mm slice thickness, and 3D models of the coronary artery were created with a CT work station to check the perfusion. Supplemental perfusion was carried out until the terminal coronary arteries appeared clear, especially for the arteries in the interventricular septum. After that, the hearts were fixed in 10% formalin for 2 weeks.

The data sets of Dicom images of the heart specimens were imported into Mimics 15.0. The processing functions of Mimics 15.0, such as “Threshold”, “Region growth” and “Edit”, were used to segment the contours of the coronary artery and the heart tissue to calculate the 3D models, which were assigned different colors.

Based on previous studies, the nutritious artery of the SAN is the sinuatrial node artery (SANA)^15–20^; the AVN is mainly supplied by the atrioventricular node artery (AVNA)^16, 20–22^ and can sometimes also be supplied by the first septal artery^21, 23^; and the nutritious arteries of the HBB are the AVNA and the first septal artery of the left anterior descending artery^24^. The nutritious arteries of the RBB are the AVNA and the septal arteries^19, 24^. The LBB is dually supplied by the AVNA and the septal arteries^24^.

The nutrient arteries of the CCS were first identified on the model of the coronary artery, and then the CCS was located.

## Location and dissection of the CCS

### Location and dissection of the SANA and SAN

**Figure 6:**
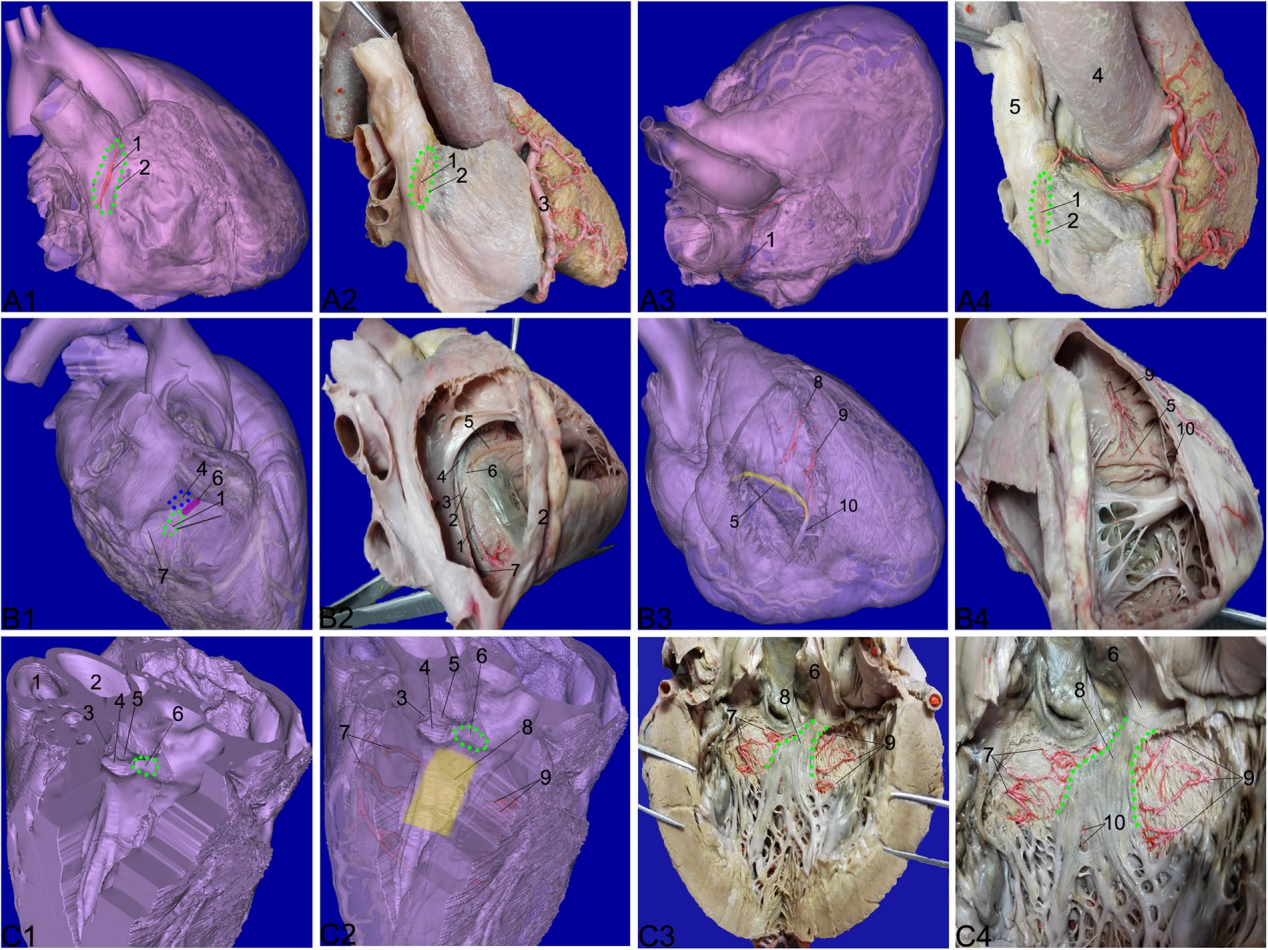
The 3D model and gross specimen. **A1** 3D model of the 1^st^ sample. The SANA (in red) arises from the RCA and crosses the sulcus terminalis, penetrating the SAN (green circle). The SAN is located at the junction of the superior vena cava with the right atrium. **A2** Corresponds to **A1** and is the gross specimen of the 1^st^ sample. **A3** and **A4** Viewed from the base to display the course of the SANA (1 SANA; 2 SAN; 3 RCA; 4 aorta; 5 superior vena cava). **B1** 3D model of the 2^nd^ sample. The AVNA (in red) arises from the posterior interventricular branch of the right coronary artery, penetrating the atrioventricular junction. The AVN (green circle) is at the apex of the triangle of Koch. The HBB (purple region) is situated below the atrioventricular part of the membranous part (blue circle). **B2** Corresponds to **B1** and is the gross specimen of the 2^nd^ sample. **B3** 3D model of the 2^nd^ sample. The RBB (yellow region) goes towards the right side of the interventricular septum at the crest, penetrating the moderator band. Its nutritious artery is the septal artery (in red). **B4** Gross specimen of the 2^nd^ sample and corresponds to **B3** (1 AVNA; 2 AVN; 3 the tendon of Todaro; 4 atrioventricular part of the membranous septum; 5 RBB; 6 HBB; 7 opening of the coronary sinus; 8 first septal artery; 9 second septal artery; 10 moderator band). **C1** and **C2** 3D model of the 3^rd^ sample. The LBB (yellow region) is situated at the lower rim of the membranous septum (green circle) and is supplied by both the anterior and posterior septal arteries (in red). **C3** and **C4** Gross specimen of the 3^rd^ sample was dissected with reference to the 3D model of the 3^rd^ sample (1 pulmonary trunk; 2 aorta; 3 right semilunar cusp; 4 left semilunar cusp; 5 posterior semilunar cusp; 6 membranous septum; 7 anterior septal arteries; 8 LBB; 9 posterior septal arteries; 10 nutritious arteries of LBB).

From the 3D model of the coronary artery (Fig. 6A1,A3), we know that the SANA arises from the right coronary artery. It travels upwards and then to the right on the anterior surface of the right atrium, goes towards the superior vena cava and circles its orifice counterclockwise, and then reaches the SAN. The sinuatrial region that we located was 21 mm in length and 4 mm in width, situated at the junction of the superior vena cava with the right atrium (Sánchez-Quintana^6^ reported that the mean length of the SAN is approximately 13.5mm (range 8–21.5 mm), being >16 mm in 50% of the hearts. The width is between 2 and 3 mm^25^). We extended the region of the node in order to make sure the node was in the area.

The SANA was dissected cautiously, and the epicardium was removed over the SAN, using the guide of the 3D model of the nutritious artery of the SAN (Fig. 6A2,A4).

### Location and dissection of the AVNA and AVN

Using the 3D model (Fig. 6B1), we could learn the course of the AVNA. The AVNA arises from the posterior interventricular branch of the right coronary artery and then penetrates the atrioventricular junction. The atrioventricular region that we located at the apex of the triangle of Koch was 8 mm in length and 6 mm in width (Jerrold Widran reported that the length of the AVN is between 5 and 7 mm and the width is between 2–5 mm^26^).

First, using the 3D model, we found the AVNA at the posterior-interventricular sulcus and then traced the artery into the atrioventricular area approximately 5 mm. Second, along the Eustachius’s valve and the Besiud’s valve, we dissected the tendon of Todaro. Third, we dissected the triangle of Koch and then removed the endocardium and the fat tissue in the area. Finally, tracing the AVNA, we dissected the AVN at the base of the atrial septum at the apex of the triangle of Koch (Fig. 6B2).

### Location and dissection of the HBB

Using the 3D model (Fig. 6B1), we identified the first septal artery of the left anterior descending artery. The area of the HBB that we located in the 3D model was 3 mm in width and 3 mm in length and was situated below the atrioventricular part of the membranous part (Jerrold Widran^22^ reported that the width of the HBB is between 1 and 2.5 mm).

The HBB could be identified by relying on its continuance from the AVN that had been dissected above. Along the atrioventricular node tail, we could find some muscle fibers continued from the tail and removed the dense connective tissue of the right fibrous trigone over the muscle fibers by using tweezers. At the same time, we dissected the first septal artery of the left anterior descending artery using the 3D model. After distinguishing the muscle fibers from the surrounding tissue, we could find the muscle fibers situated around the portion of the membranous part of the interventricular septum (Fig. 6B2).

### Location and dissection of the RBB

Using the 3D model (Fig. 6B3), we could learn the course of the AVNA and the first and second septal arteries of the left anterior descending coronary artery. Taking into consideration the nutritious arteries and course and dimension of the RBB (the RBB ramifies from the HBB at the anterior-inferior side of the membranous septum and then goes towards the right side of the interventricular septum at the crest, finally to the moderator band; the RBB is 1 mm in thickness^27^), we located the RBB in the 3D model.

We dissected the second septal artery of the left anterior descending coronary artery. As the RBB ramifies from the HBB, we traced the muscle fibers that continued from the HBB. The second septal artery of the left anterior descending coronary artery has one branch running upwards to supply the upper 1/3 of the RBB with the other branch irrigating the inferior 2/3, accompanying the RBB to penetrate the moderator band (Fig. 6B2,B4).

### Location and dissection of the LBB

Per the 3D model (Fig. 1C1,C2), we could learn that the left main bundle branch is supplied by both the anterior septal arteries and the posterior septal arteries. The left main bundle branch that we located was situated at the lower rim of the membranous septum and was 10 mm in width and 30 mm in length (George K. Massing^27^ reported that the width of the LBB origin is from 2 to 14 mm and the length is 35 mm).

We traced the nutritious arteries to the region using the 3D model and then removed the endocardium over the region with tweezers (Fig. 6C3,C4).

### Histological and immunofluorescence examination

The histological criteria we chose were suggested at the meeting of the German Pathological Society held at Erlangen in 1910. First, the cells making up the proposed tracts should be histologically distinct from the adjacent myocardium. Second, it should be possible to trace the tract of the CCS through serial sections. Third, and most importantly, the cells within the tract should be insulated from the neighboring myocardium by fibrous sheaths^28^.

Simon J. Crick^29^ and Louis Tsun Cheung Chow^30^ reported that PGP9.5 was profuse in the CCS, displaying a significantly higher density of PGP9.5 in the CCS than the adjacent myocardium. Louis Tsun Cheung Chow^30^ also found that TH was expressed more abundantly in the CCS than the myocardium. Hyperpolarization-activated cyclic nucleotide-gated channel 4 (HCN4), the principal ion channel responsible for the funny current (If), an important pacemaker current in the SAN and AVN, is known to be expressed in the atrioventricular rings (AV rings)^31^. Immunohistochemistry showed that the Connexin 40 (Cx40) protein was highly abundant in the compact node (CN) but absent from the ventricular muscle (VM). What is more, immunohistochemistry showed that the Cx40 protein was more abundant in the penetrating bundle/bundle of His (PB) than in the working myocardium^32^. Therefore, in this study, we used anti-PGP9.5, anti-TH, anti-Cx40 and anti-HCN4 antibodies to perform the immunofluorescence examination.

The structures that were dissected using the 3D model were cut from the hearts, and then these tissues were subsequently rinsed in phosphate-buffered saline (PBS, 0.01 M, pH 7.2–7.4, Salarbio). Tissues embedded in paraffin were serially sectioned at 5-μm thickness and stained with hematoxylin-eosin and Masson’s trichrome as well as immunofluorescence staining.

For the immunofluorescence staining, the paraffin-embedded dewaxed sections were incubated with anti-protein gene product 9.5 (PGP9.5, 1:500, Millipore) and the goat anti-rabbit IgG-488 (1:400, Life) secondary antibody. The paraffin-embedded dewaxed sections were incubated with anti-tyrosine hydroxylase (TH, 1:500, Millipore) and the goat anti-rabbit IgG-488 (1:400, Life) secondary antibody. The paraffin-embedded dewaxed sections were incubated with anti- hyperpolarization-activated cyclic nucleotide-gated channel 4 (HCN4, 1:500, Abcam) and the goat anti-rabbit IgG-488 (1:400, Life) secondary antibody. The paraffin-embedded dewaxed sections were incubated with anti-connexin 40 (Cx40, 1:500, Abcam) and the goat anti-rabbit IgG-488 (1:400, Life) secondary antibody.

## Electronic supplementary material

Supplementary information
