# Supplementary material for: Locating the Human Cardiac Conduction System Using a 3D Model of Its Nutritious Arteries
Source: Sci Rep. 2017 Mar 23;7:344. doi: 10.1038/s41598-017-00504-1 (PMC5428715; doi:10.1038/s41598-017-00504-1)
Supplement: Supplementary file 1 — Supplementary information [file 41598_2017_504_MOESM1_ESM.pdf]

Supplementary information

Locating the Human Cardiac Conduction System Using a 3D Model of Its Nutritious Arteries

Yu Xu[1△], Yukun Peng[1△], Rongmei Qu [2△], Guorong Zheng [3], Feiyan Feng[1], Yan Feng4, Linying He[5], Shanli He[2], Zeyu Li[2], Chang Liu[2], Zhaoming Xiao[2], Jun Ouyang[2☆], Jingxing Dai[2☆]

1 First Clinical School, Southern Medical University, Guangzhou, China,

2 Department of Anatomy, Guangdong Provincial Medical Biomechanical Key Laboratory, Southern Medical University, Guangzhou, China,

3 Department of Radiology, Guangdong No.2 Provincial People's Hospital, Guangzhou, China,

4 Department of Electrocardiogram, Xinjiang Uygur Autonomous Region People's Hospital, Urumqi, China,

5 Second Clinical School, Southern Medical University, Guangzhou, China.

[△] Yu Xu, Yukun Peng, Rongmei Qu equally contributed to this article.

[☆] **Corresponding authors: Jun Ouyang**, Ph.D., Department of Anatomy, Guangdong Provincial Medical Biomechanical Key Laboratory, Southern Medical University, Guangzhou, 510515, China, Email: jouyang@126.com, Tel: +86(20)61648199; or **Jingxing Dai**, Ph.D., Department of Anatomy, Guangdong Provincial Medical Biomechanical Key Laboratory, Southern Medical University, Guangzhou, 510515, China, Email: daijx2013@163.com, Tel: +86(20)61648198.

Table 1. The rate of permanent pacemaker implantation

Author	Year	Transfemoral(TF)※	Transapical(TA)※	Transaortic(TAO)※
Raj R. Makkar[1]	2012	4.7%Δ	NR	NR
David M. Holzhey[2]	2012	NR	25/167(15.0%)+	NR
Giuseppe Bruschi[3]	2012	NR	NR	4/25(16.0%)
Joel A. Lardizabal[4]	2013	NR	5/76(7.0%)+	NR
Giuseppe Bruschi[5]	2013	NR	NR	7/47(14.8%)
Kanstantions Spargias[6]	2014	NR	NR	3/25(12.0%)
Ian T. Meredith AM[7]	2014	34/119(28.6%)+	NR	NR
Eugene H. Blackstone[8]	2015	78/1191(6.5%)+	78/1191(6.5%)+	NR
Corrado Tamburino[9]	2015	15.5%	NR	NR
Katie E. O' Sullivan[10]	2015	NR	7.4%	6.1%

※Values presented n/N(%) or %＋Clinical outcomes at 30 days Δ Clinical outcomes at 1 year

Table 2. The rate of aortic valve regurgitation

Author	Year	Transfemoral(TF)※	Transapical(TA)※	Transaortic(TAO) ※
Raj R. Makkar[1]	2012	18/144(12.5%)+	NR	NR
Jorg Kempfert[11]	2012	NR	13/299(4.3%)	NR
Joel A. Lardizabal[4]	2013	NR	9/76(12.0%)	5/44(11.0%)
Kevin L. Greason[12]	2014	11/92(12.0%)	8/95(8.4%)	NR
Eugene H. Blackstone[8]	2015	54/441(12.2%)	34/413(8.2%)	NR
Corrado Tamburino[9]	2015	9.8%	NR	NR
Sa R kapadia[13]	2015	23/165(14.0%)	NR	NR

※Values presented n/N(%) or %＋Clinical outcomes at 30 days Δ Clinical outcomes at 1 year

References:

[1] Makkar RR, Fontana GP, Jilaihawi H, Kapadia S, Pichard AD, Douglas PS, *et al.*. Transcatheter aortic-valve replacement for inoperable severe aortic stenosis. N Engl J Med 2012;366:1696-1704.

[2] Holzhey DM, Shi W, Rastan A, Borger MA, H Nsig M, Mohr FW. Transapical versus Conventional Aortic Valve Replacement? A Propensity-Matched Comparison. The Heart Surgery Forum 2012;15:4-8.

[3] Bruschi G, de Marco F, Botta L, Cannata A, Oreglia J, Colombo P, *et al.*. Direct aortic access for transcatheter self-expanding aortic bioprosthetic valves implantation. ANN THORAC SURG 2012;94:497-503.

[4] Lardizabal JA, O'Neill BP, Desai HV, Macon CJ, Rodriguez AP, Martinez CA, *et al.*. The Transaortic Approach for Transcatheter Aortic Valve Replacement. J AM COLL CARDIOL 2013;61:2341-2345.

[5] Bruschi G, Botta L, De Marco F, Colombo P, Nonini S, Klugmann S, *et al.*. Direct aortic transcatheter valve implantation via mini-thoracotomy using the Medtronic CoreValve. Multimedia Manual of Cardio-Thoracic Surgery 2013;2013:t15.

[6] Spargias K, Bouboulis N, Halapas A, Chrissoheris M, Skardoutsos S, Nikolaou J, *et al.*. Transaortic aortic valve replacement using the Edwards Sapien-XT Valve and the Medtronic CoreValve: initial experience. Hellenic J Cardiol 2014;55:288-293.

[7] Meredith AI, Walters DL, Dumonteil N, Worthley SG, Tchetche D, Manoharan G, *et al.*. Transcatheter aortic valve replacement for severe symptomatic aortic stenosis using a repositionable valve system: 30-day primary endpoint results from the REPRISE II study. J AM COLL CARDIOL 2014;64:1339-1348.

[8] Blackstone EH, Suri RM, Rajeswaran J, Babaliaros V, Douglas PS, Fearon WF, *et al.*. Propensity-matched comparisons of clinical outcomes after transapical or transfemoral transcatheter aortic valve replacement: a placement of aortic transcatheter valves (PARTNER)-I trial substudy. CIRCULATION 2015;131:1989-2000.

[9] Tamburino C, Barbanti M, D'Errigo P, Ranucci M, Onorati F, Covello RD, *et al.*. 1-Year Outcomes After Transfemoral Transcatheter or Surgical Aortic Valve Replacement: Results From the Italian OBSERVANT Study. J AM COLL CARDIOL 2015;66:804-812.

[10] O' SK, Hurley ET, Segurado R, Sugrue D, Hurley JP. Transaortic TAVI Is a Valid Alternative to Transapical Approach. J Card Surg 2015;30:381-390.

[11] Kempfert J, Rastan A, Holzhey D, Linke A, Schuler G, Mohr FW, *et al.*. The learning curve associated with transapical aortic valve implantation. Ann Cardiothorac Surg 2012;1:165-171.

[12] Greason KL, Suri RM, Nkomo VT, Rihal CS, Holmes DR, Mathew V. Beyond the learning curve: transapical versus transfemoral transcatheter aortic valve replacement in the treatment of severe aortic valve stenosis. J Card Surg 2014;29:303-307.

[13] Kapadia SR, Leon MB, Makkar RR, Tuzcu EM, Svensson LG, Kodali S, *et al.*. 5-year outcomes of transcatheter aortic valve replacement compared with standard treatment for patients with inoperable aortic stenosis (PARTNER 1): a randomised controlled trial. LANCET 2015;385:2485-2491.

Figure S1. ACHE staining of cardiac conduction system (CCS). A, SAN. B, AVN. C, HBB and RBB. D, LBB (OLYMPUS IX83, scar bar=150μm).

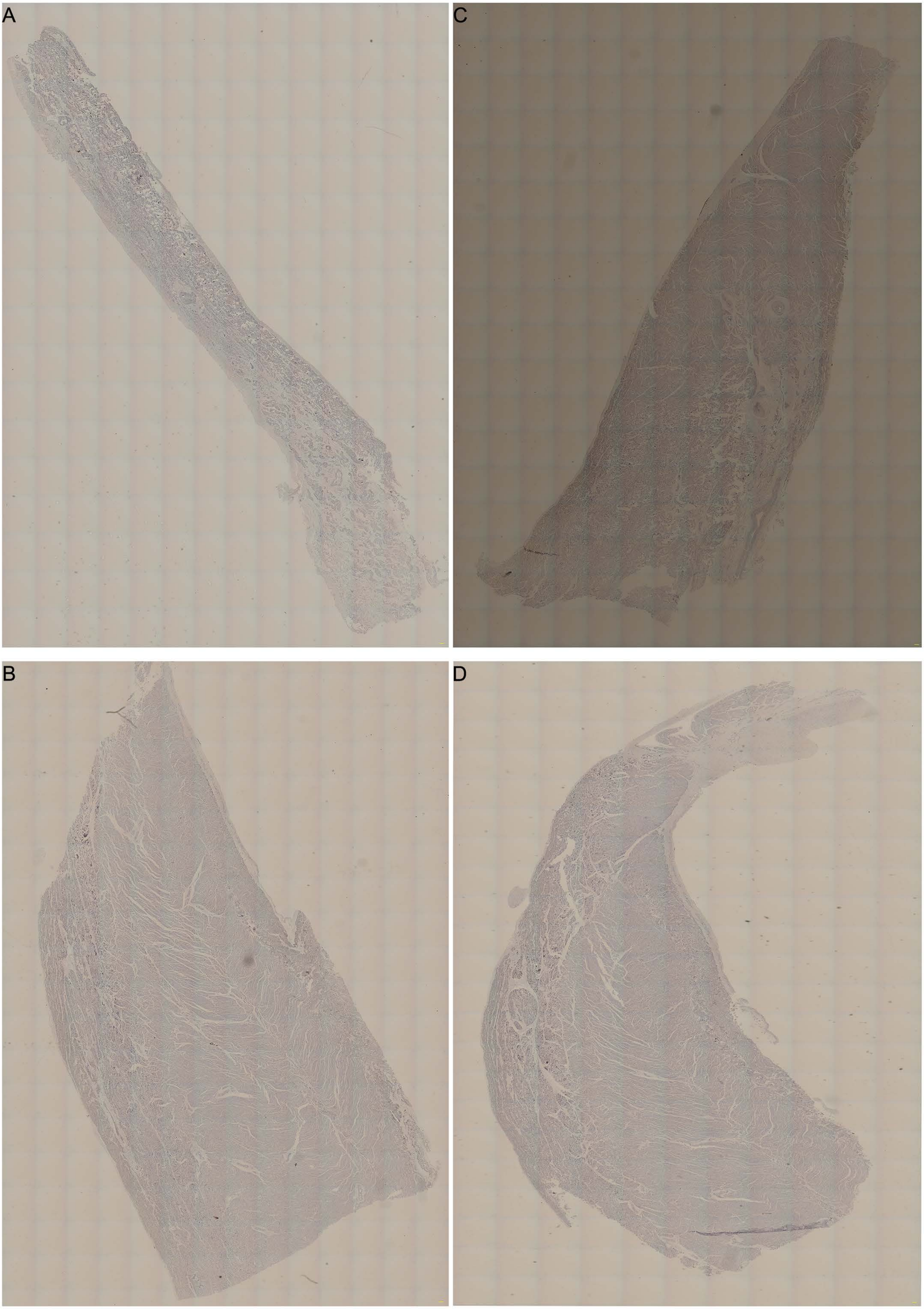
A
C
B
D
